# The modelling cycle for collective animal behaviour

**DOI:** 10.1098/rsfs.2012.0031

**Published:** 2012-08-15

**Authors:** David J. T. Sumpter, Richard P. Mann, Andrea Perna

**Affiliations:** Mathematics Department, Uppsala University, Uppsala, Sweden

**Keywords:** collective animal behaviour, theoretical modelling, collective motion

## Abstract

Collective animal behaviour is the study of how interactions between individuals produce group level patterns, and why these interactions have evolved. This study has proved itself uniquely interdisciplinary, involving physicists, mathematicians, engineers as well as biologists. Almost all experimental work in this area is related directly or indirectly to mathematical models, with regular movement back and forth between models, experimental data and statistical fitting. In this paper, we describe how the modelling cycle works in the study of collective animal behaviour. We classify studies as addressing questions at different levels or linking different levels, i.e. as local, local to global, global to local or global. We also describe three distinct approaches—theory-driven, data-driven and model selection—to these questions. We show, with reference to our own research on species across different taxa, how we move between these different levels of description and how these various approaches can be applied to link levels together.

## 1. Introduction

All biological systems have complex organization at different levels: molecules interact to form subcellular organules, cells organize together to produce tissues and so on. Even if we are aware that this complexity exists, most of the time we do not experience it directly with our senses. The interactions take place at spatial and temporal scales that are either too short or too long for us to grasp. But there is one level at which the complexity of biological interactions is immediately apparent to our eyes. It is the level of collective animal behaviour. When we observe a group of starlings coming together and flying in the evening light, a bank of fish attacked by a predator, a line of marching ants, we see the individual animals moving and interacting, and at the same time, we notice the appearance of another level of order of a different kind: the order of the flock, of the school, of the ant colony.

Understanding the behaviour of animal groups does not require a complete understanding of the behaviour of individual animals. Consider an everyday example of car traffic. Every rush hour, the patterns of congestion that form depend strongly on the density of vehicles, but weakly if at all on the origins or final destinations of the drivers. The same is true at all levels of physical, biological and social organization. Experiments and theoretical studies show that similar patterns of collective behaviour emerge in systems as varied as humans, animals, tissue cells and microorganisms [1–3]. Often there is a characteristic dependence on the density of individuals, but the patterns created are independent of the specific identities of these individuals. We do not need to know the individual histories and motivations of hundreds of individuals to predict what the group as a whole will do.

On the other hand, individual motivations and interactions are often the object of interest in studies of animal behaviour. We are interested in understanding the basic behavioural cues followed by and the survival strategies adopted by individuals, even when our ultimate aim is to better understand group behaviour [4,5]. Investigation of these cues can take a wide range of forms, from looking at how optic flow can be used to measure distance [6] and avoid collisions [7] to testing hypotheses about optimal foraging decisions [8]. These and many other studies form the core of animal behaviour research, providing basic information about how individuals react to stimuli and interact with each other.

Creating a dichotomy between understanding the group through simplified assumption about individual behaviour and detailed quantification of how individuals interact can be an extremely useful approach in understanding collective animal behaviour. Ultimately, however, the research goal is to find mutual relations between these two levels of description. This point is especially relevant when looking at collective motion or spatial structures created by animal groups. In such situations, the behaviour of even large groups can be drastically altered by manipulating the strength of interactions and the movement of just a fraction of individuals [9,10]. If we understand the nature of these interactions we can conceivably change them to control the group behaviour [11,12].

New technology, such as GPS [13], radio-frequency identification tags [14], three-dimensional image analysis [15,16] and radio tracking [17], means that we now have access to large quantities of data at both the individual and global levels. Precisely because collective animal behaviour occurs on temporal and spatial scales that we can relate to, means that it has become possible to automate tracking of interactions. This gives collective animal behaviour research a head start on many other areas of complex systems research, but it also means that we need to develop a clear framework within which to analyse, quantify and make use of these data.

In this study, we provide a basic classification of the different levels at which collective animal behaviour is studied. We also present three modelling methodologies that can be applied, which we call theory-driven, data-driven and model selection. We then use this framework to review case studies in phase transitions, collective structures and collective motion. Our focus is on the degree to which we can complete the modelling cycle for such systems. To what extent can we go from observing global patterns, to using simple models to describe plausible local interactions between individuals, to then empirically quantify these interactions, and finally predict and check consistency of the empirical rules with the global patterns?

## 2. Methodological approaches

In some cases, individual behaviour is less important than understanding groups, and in other cases, groups can be largely ignored when studying individuals. It is worth then pausing to evaluate and classify the level of description on which different research projects operate. We use the following classification.

*Local*. Here, we study and make predictions about the movements or behaviour of individuals on the basis of the local configuration of the environment and other individuals around the animal. In analogy to physics, this can be thought of as the ‘classical mechanics’ approach.
*Local to global*. Here, we aim to directly link the behaviour of individuals to the classes of patterns produced by groups. This is the question of studying self-organization in biological systems [3]: to what extent can simple interactions produce interesting collective patterns? In almost all cases, the step from local to global is carried out with the help of a mathematical model, where simplifying assumptions have been made about how individuals interact. In physics, this approach is often referred to as ‘kinetic theory’ and sometimes as a subset of ‘statistical mechanics’.
*Global*. The focus is entirely on the large-scale macroscopic pattern. Here, a theory about the evolution of a large-scale structure tries to predict how it will change in time or react to perturbations. This is a purely ‘statistical mechanics’ approach, where we describe ensembles of individuals.
*Global to local*. Here, we identify regularities in the group configuration or dynamics and use these to propose possible local rules of interaction between individuals. The global patterns place restrictions on the set of possible local interactions. This is often referred to as the ‘inverse problem’.

In what follows, we give a number examples of studies falling in to each of these classes.

A defining feature of the study of collective animal behaviour is the interplay between experiment and theoretical models. Most research aims at either deriving or testing a model for how individuals interact to produce collective patterns. We classify modelling approaches in three ways.

*Theory-driven approaches*. Here, the process starts from models that in their first formulation have been only informally constrained by data. Theory-driven approaches are usually based on a model that predicts the large-scale properties from a set of local interactions (local to global). Only in a next stage are data used to support or refute the theory.
*Data-driven approach*. Under this approach, we start from the position of analysing and making sense of experimental or observational data. We try to identify consistent patterns, but only with informal use of models. This process is often either purely local, where experimental data are used to quantify the response of individuals to the stimuli that they encounter around themselves, or purely global, where the shape and structure of groups are quantified.
*Model selection approach*. This approach does not insist on the production of models either from data or from theory, but on their evaluation. Starting from a set of data and different models, it aims to quantify how closely each model approximates the data. The aim is to determine to what degree one model is preferable to another and search for better alternatives.

The theory-driven, data-driven and model selection approaches are complementary. They are all combined within a *modelling cycle*, where we move up and down between the local and global levels. This movement between levels imposes a structure on the modelling cycle. We now describe the character of this process, using several case studies.

## 3. Phase transitions

One of the original theory-driven models in the study of collective motion is the self-propelled particle model proposed by Vicsek and co-workers [18–20]. This model is local to global. Each particle adjusts its own direction to be nearer to the average direction of the particles within a local radius. At the global level, as the number of particles increases, there is a phase transition from disordered movement to collective order and organized motion, with spontaneous direction changes observed near the transition point. This prediction was confirmed in marching locusts placed in an experimental ring [21] and then later in field observations [22]. The prediction of the Vicsek model is ‘universal’, in the sense that the same global pattern emerges from differing underlying rules [2] (although see Gregoire *et al*. [23] for a discussion of how rules determine outcome). Indeed, not just locusts, but a large number of animal species all reproduce the same transition from random to aligned motion [24–26].

The fact that individual locusts interact locally supports the basic assumptions of the Vicsek model [21,27]. However, the match between theory and experiments is primarily at the global level. These studies do not identify or quantify the cues and responses used by animals when interacting with each other or with their environment. In order to provide a local description of interactions, Bazazi *et al*. [28] manipulated the sensory inputs to locusts, finding that visual and tactile stimuli originating from behind the locust were those that resulted in collective motion. Marching bands of locusts and crickets may form because individuals are attempting to eat those in front of them and get away from those behind [29]. Protein hungry locusts and crickets cannibalize conspecifics, and collective motion may result from a ‘forced march’ [30,31]. On this basis of local mechanism (i.e. cannibalism) and a global outcome (i.e. collective motion), these researchers built a model to link these two levels. In the model, they hypothesized that hunger increases individual interaction strengths and showed that this assumption was sufficient to explain increased marching speed of hungry locusts [32].

While showing that a local-level model is sufficient to explain global-level data is essential, we are often interested in comparing multiple models and finding which one provides the best explanation. This point is particularly relevant when so many models can produce the same global-level outcome. It is here that model selection can be applied. In order to investigate how model selection might be used in collective animal behaviour, we looked at interactions of glass prawns [33]. Compared with the many kilometre wide marching bands created by locusts, the collective patterns made by these prawns are much less spectacular. The prawn interactions are also simpler, primarily mediated by tactile contacts. This simplicity makes them an ideal test bed for model selection.

**Figure 1.**
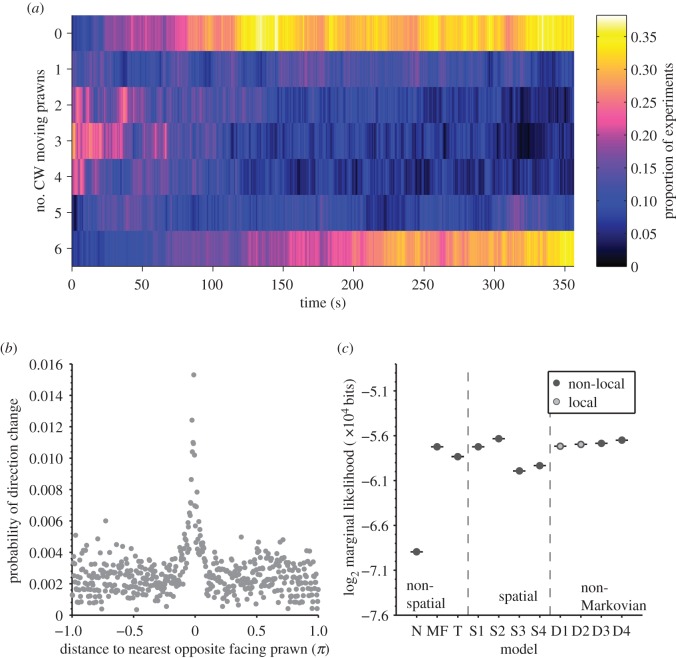
Analysis of collective behaviour in glass prawns. (*a*) The proportion of experiments with a given number of prawns travelling clockwise (CW) over time. This exhibits a phase transition from an initially disordered state, where most experiments have roughly half the prawns travelling CW, to aligned collective motion in either a CW or a counter-CW direction. (*b*) The probability that a prawn changes its direction of motion as a function of the position of the nearest conspecific travelling in the opposite direction. Prawns are more likely to change direction when close to another individual moving in the opposite direction. (*c*) Model selection favours either Markovian spatial models with large interaction radii (non-local interactions) or non-Markovian models with a local interaction. The model with the highest likelihood is a Markovian model with a large interaction zone (*π* radians). The only models with local interactions (<*π*/2 radians) are two non-Markovian models with memory effects, D1 and D2.

The prawns were put in a ring-shaped arena (external diameter: 20 cm) and filmed for 6 min. At the global level, we found that the prawns exhibited a similar phase transition as in the Vicsek model (figure 1*a*). We also found that prawns increased their probability of changing direction when they had a prawn nearby (figure 1*b*). We then confirmed that the global pattern could be reproduced accurately by a variety of appropriately parametrized models; some, such as the Vicsek model, involved only local interactions, whereas others included memory of previous encounters and global interactions. To determine which model fits the data best, we first translated the rules of motion in each model into a probability that a focal prawn will change its direction of motion based on either the local or global configuration of other individuals around it. By assessing this probability against the actual observed events (either the focal prawn changes direction or it does not), we were allowed to calculate the *likelihood* for each model, i.e. the probability of the complete data sequence of movements over the duration of the experiments conditioned on the model being examined. This process also gave us the estimate of the model parameters. To check the consistency of these parametrized models with patterns of direction changes at the global level, we then simulated the models with parameters estimated, and compared these with the collective motion of larger groups of prawns. Through this process, we could establish the existence of either non-local interactions or a memory of previous interactions. The likelihood of various models is ranked in figure 1*c*. The model with the highest likelihood is Markovian and has a very large (∼*π* radians) interaction zone. This interaction zone is a non-local effect—it extends far beyond the plausible sensory perception of the prawn itself and is inconsistent with the localized effect seen in figure 1*b*. Of those models whose likelihood exceeds the mean field model, only non-Markovian models allow for a *local* interaction range consistent with figure 1*b* (∼*π* /5 radians). Simulation of these non-Markovian models confirms that they reproduce the group level alignment seen in figure 1*a*, which represents a necessary large-scale condition. The prawn study illustrates the importance of distinguishing sufficiency and necessity in model fitting. A large number of models were sufficient to explain the data, but the memory of interactions was necessary to retain consistency between descriptive levels and with biological intuition.

## 4. Collective structures

Sometimes, the behaviour of animal groups results in the formation of physical structures: the dams collectively built by beavers, the mounds of termites and the networks of galleries of naked mole rats [34]. One such structure is the pattern of trails collectively produced by animals and humans [3,35,36]. The formation of trails is in many respects analogous to a ‘phase transition’ from random to coherent state. In this case, the transition is not in alignment, but in the spatial distribution of the individuals. A simple illustration of such a transition is the double bridge experiment, where a colony of ants are presented with a branching bridge with the two branches leading to different food sources. As the flow of ants from the nest increases we go through two transitions. First, from a disorganized state where branches are chosen at random to a situation where a pheromone trail forms to just one of the food sources [37,38], then a transition from one to two trails as the food sources become crowded [39].

These transitions are captured by differential equation models of the number of ants going to each feeder as a function of flow rate. The underlying assumptions of these models are that the ants respond nonlinearly to pheromone concentration, and positive feedback determines which food source is selected [40–42]. Such models are global in the sense that they provide a mean-field model of trail formation: in the models pheromone concentrations are updated, depending on the average probabilities of choosing one branch or the other. A number of theory-driven local to global models of trail formation have been proposed to explain the formation of trail networks [35,43–46]. These simulations reproduce the bifurcating structure seen in ant pheromone trails, again using unquantified, but biologically realistic, assumptions about how ants leave pheromone and react to it.

**Figure 2.**
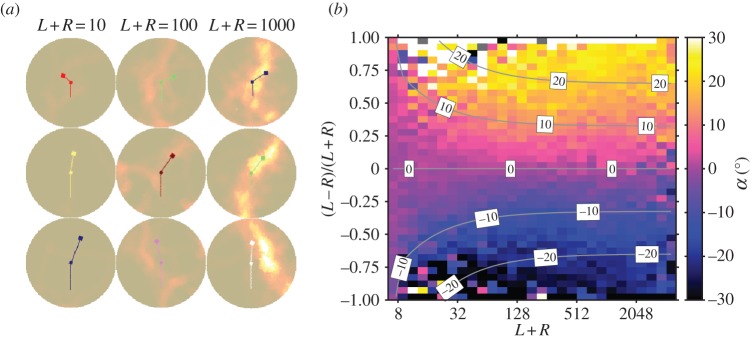
Ant response to pheromone concentrations. (*a*) Individual observations of ant movement on top of the ‘pheromone map’ produced by their own colony. Brighter colours indicate higher pheromone concentrations or, equivalently, more ant passages. The trajectory of the focal ant is summarized by its position at three consecutive time intervals of 400 ms each. Both the pheromone map and the ant's trajectory are rotated to be aligned together. The maps can be grouped together based on the amount of pheromone present in front-left (*L*) and front-right (*R*) sectors around the ant. (*b*) Turning angle (*α*) of the ant as a function of pheromone concentrations. The solid lines are the turning angles predicted by equation (4.1) with *A* = 30.80 and *T*_0_ = 10.53. When the total pheromone concentration is low, ants' turning angle is less correlated with concentrations, but for higher concentrations the turning angle is proportional to (*L* − *R*)/(*L* + *R*).

We adopted a data-driven approach to characterize the actual response of ants to pheromone concentrations [47]. We estimated pheromone concentrations by recording the passages of ants at all spatial positions in an experimental arena. These observations allowed us to infer a ‘pheromone map’ where we could look at correlations between pheromone concentrations and changes in speed or direction of movement of the ants (figure 2*a*). The ant turning angle *α* was a function of pheromone differences such that4.1
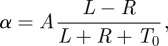
where *L* and *R* are the concentrations of pheromone on the left and on the right side of the ant. *A* is a constant and *T*_0_ is a threshold below which the ants cannot effectively detect pheromone. Such an individual response is the same as seen in human perception of sensory stimuli, such as sound loudness, image brightness, time, numerosity and weight [48]. This type of response, where the perception of a difference between two stimuli is inversely proportional to the magnitude of the stimuli, is known as Weber's Law [49].

Figure 2*b* shows how turning angle changes as a function of stimuli difference and total stimuli intensity, comparing experimental observations with equation (4.1). Here, the pattern of how response changes with pheromone concentration helps explain the outcome of the model of the transition from disorganized foraging to a choice of one of the feeders in the double bridge experiment. However, equation (4.1) is essentially linear, while a nonlinear response to pheromone was required to produce this transition: trails can only be reinforced if the ants have a disproportionally higher probability to select the trail with higher pheromone concentration [42]. To reconcile these local and global levels, we showed that when integrating over repeated interactions between the ants and the pheromone trail, an appropriate nonlinearity appears, which explains the first of these transitions [47].

The data-driven approach allowed us to establish empirically how individual ants respond to pheromone and reconcile this with some aspects of their collective behaviour. However, when we implemented a spatially explicit simulation of trail formation in which ants follow Weber's Law, we found some differences between the exploratory pattern formed by the simulated ants and that formed by the real ants. In particular, simulated ants built more reinforcing loops that remain stable for indefinite time. These results suggest that some additional navigation ability such as path integration or response to other ants may be required to model exploratory network formation [50].

## 5. Collective motion

The self-propelled particle models used to explain phase transitions can be extended to capture other aspects of collective motion. Models that include repulsion, attraction and alignment responses to neighbours reproduce many of the dynamic features of bird flocks and fish schools [51–58]. These models produce swarms, rotating tori, dynamic moving groups with continually changing internal structures as well as many other aspects of the movement of animal groups. Although these models may look reasonable at a global level, the question remains as to which (if any) of these models best captures what the animals actually do.

The first stage in linking these models to data is quantifying the global shape and dynamics of flocks and schools. This is non-trivial, because the movements are highly complex and usually occur in three dimensions. Despite these challenges, basic information about the structure, shape and dynamics of starling flocks [15,59,60] and fish schools [61] has now been established. It may be possible to use some of these data to solve the inverse problem of going from global structure to local interactions. For example, the topological arrangement of neighbouring starlings is largely independent of flock density, suggesting that starlings interact with a limited set of nearest neighbours, rather than interacting with all neighbours within a fixed distance [15]. However, a local to global model in which individuals interact with neighbours in inverse proportional to distance between them also reproduces the same topological arrangement [55]. This modelling exercise again confirms that if we are really interested in evaluating and comparing alternative self-propelled particle models, we need at least some experimental information about how individuals interact locally.

**Figure 3.**
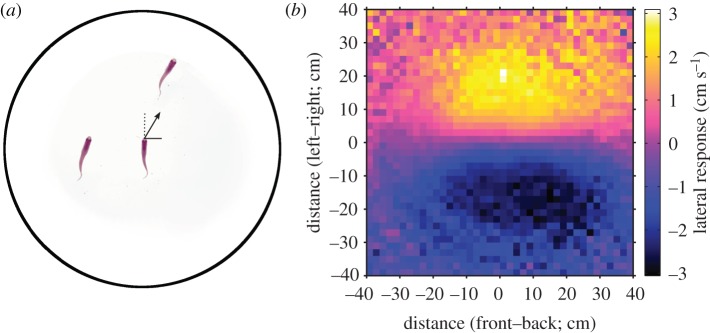
Fish response to their neighbours. (*a*) Snapshot of a shoal of mosquitofish seen from above. The focal fish (in the centre) responds to the neighbours by changing its speed and direction of movement. The lateral component of the response indicated by the solid line is plotted in (*b*) as a function of the position of the neighbours.

Two recent studies have looked at local interactions between fish using a data-driven approach [62,63]. The rules of interaction were determined by identifying correlations between the relative positions and directions of individuals and the response of a focal individual, in terms of changes in direction or speed. By investigating how these responses vary as a function of the configuration of the neighbouring individuals, the interaction rule can be observed directly. For example, figure 3 plots the left–right component of the movement of the focal fish in response to the position of its neighbours (its ‘lateral response’ in figure 3*b*). The response is such that the focal fish generally turns towards the neighbours, and collision avoidance is mainly mediated through changes of speed [63].

The challenge in this approach lies in identifying which of many correlated variables is most important in predicting fish motion. For example, the acceleration of a focal fish is correlated both with the average position of all its neighbouring fish and with the position of its nearest neighbour, which are themselves correlated variables. Which of these correlations is most relevant to predicting the fish's future movement? To answer this and similar questions, we fitted a sequence of models for each of the relevant factors. First, we determined how much of a fish's future speed and direction could be explained simply as a linear function of its recent direction and acceleration changes. Then, accounting for this autocorrelation, we fitted a neural network model of how the fish responded to its physical environment (i.e. tank walls). Then, accounting for both autocorrelation and physical environment, we fitted a further neural network model, this time for how the fish responded to its nearest neighbour. Up to this point, each additional model provided an improvement in prediction of fish movements. However, when we went on to fit a model of how the second and further away neighbours affected the fish's response, we found that these models provided no further improvements in terms of predicting the focal fish's movement. Only the position of the nearest neighbour was required to predict these movements.

While we use the word ‘predict’ in the previous paragraph to indicate whether or not including additional interactions improves our model fit, we have not applied a rigorous process of model selection to the data. Ideally, we would like to introduce one parameter at a time to the model and test, as we did using the Bayes factor with the prawns, whether this parameter improves predictive power. We have used a similar approach to infer the number and location of landmarks used by homing pigeons [64]. There we evaluated the information content of each element of the path, determined the most informative points and assessed successively whether each new proposed landmark contributed predictive value to the current model. For the fish, the large number of potential combinations of stimuli means that we cannot test them all. Ultimately, the sequence whereby we fitted models first for autocorrelation, then for walls and then for each of the neighbours is motivated by ‘biological intuition’ about how fish react to stimuli rather than by the formal rigour required by model selection. That said, in all biological applications, we must rely on biological reasoning to select the possible subset of models before we apply mathematical tools. We will return to this point in §6.

The earlier-mentioned process gives a model that maps from configuration to response, either in the form of a large look-up table based on the observations or as a neural network with a large number of parameters. We could implement this mapping as a self-propelled particle simulation where at each time step, each particle evaluates its local neighbourhood and updates its speed and direction using this mapping. This model could then be used as a check as to whether global level statistics can be reproduced, helping us further evaluate whether the model matches the available data. Such a process has however some limitations compared with the self-propelled particle models discussed in the first paragraph of this section. These models have only a small number of parameters and can be stated in a handful of equations that relate explicitly to how animals respond in different situations. We can easily change the relative strengths of these responses and judge their effect on collective patterns. When our model is based on a large number of parameters inside a neural network, it is less clear on what theoretical basis we can manipulate interaction rules.

Rigorous fitting of self-propelled particle models to data would help scale up from individual level observations to predictions of global patterns. It is, in theory, possible to test the degree to which self-propelled particle models can capture the motion of real animals. Artificial flocking trajectories generated by a simulation can then, using least squares ‘force matching’, be fitted to the model which generated the data as well as alternative, incorrect models [65]. Only a small number of observations are required in order to reject the incorrect alternative models and accept the correct model. We have demonstrated a probabilistic Bayesian approach to parameter inference and model selection, allowing comparison of models with different numbers of parameters [66]. Models that over-fit the data were rejected by this method. Parameter inference in this framework provides not only the ‘best-fit’ parameter values, but also how probable any alternative parameter sets might be.

**Figure 4.**
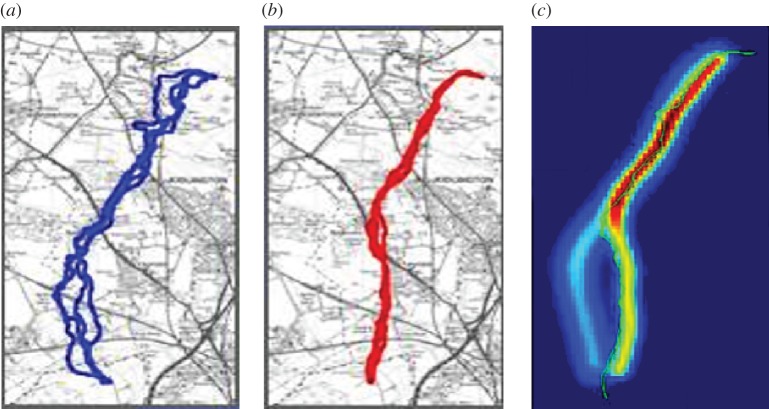
Prediction of paired pigeon flight paths from previous solo flights. (*a*) Pigeon 1 and (*b*) pigeon 2 are released repeatedly on their own from the same release site, developing idiosyncratic habitual routes back to the home loft. From the previous flights of each bird, a distribution of the likely spatial location of future flights can be determined [64]. (*c*) A weighted sum of these two distributions can predict the flight paths of each bird when they are released as a pair, with the two birds typically cooperating when the distributions overlap, and switching to either leader–follower behaviour or splitting when the distributions separate. Here, pigeon 2 leads pigeon 1 when the distributions diverge.

An alternative and complementary research direction to fitting models to data is to focus purely on predicting future outcomes without an underlying model of interactions. For example, Mann [67] looked at how pairs of pigeons decided upon a joint flight route home when flying together, based on observations of their previous preferences when flying alone (figure 4*a,b*). A distribution of each individual's flight paths was inferred from multiple solo flights [64], which then gave the probability that each individual would fly along a particular route in subsequent flights. An assumption was then made that their joint behaviour would represent a blend of these individual preferences. Collective route selection was therefore modelled by a weighted mixture of the two birds' solo distributions, with no explicit ‘rule of interaction’. Model selection was performed to determine what factors consistently affected the relative weights of the bird's own previous route and its partner's previous route. The data supported the hypothesis that a bird's degree of loyalty to its own route predicted how much it will tend to be influenced by its partner (figure 4*c*).

## 6. Discussion

There is a particular methodology to approaching collective animal behaviour that emerges from the above examples. We start by observing a global pattern and formulate a theory-driven local to global model that is sufficient to explain certain aspects of this pattern. Then, the focus switches to experimental observations at the individual level. These reveal new details of the interactions between animals, which are incorporated into new, primarily data-driven, models. When we implement these data-driven models, we again test sufficiency against the global level data, hopefully identifying additional global level properties that our new model matches.

There are two potential outcomes of this process: either the data-driven model produces the global-level pattern or it does not. Both these outcomes imply further work. If the data-driven model fails to produce the global-level pattern, then we need to reassess the individual level data or perform further experiments in order to improve our model. Here, it may be useful to try and use the global-level pattern to infer local rules. In any case, the cycle between global and local should be repeated until more is learnt about why these levels cannot be reconciled. It is worth noting that failures to reproduce global patterns are much less widely reported in the literature. This is partly because scientists repeat this part of the cycle before publishing, and then only report the model that ‘works’. Similarly, if no model works, then they might all be omitted from the paper. This, like other failures to report negative results, is an unfortunate but unavoidable limitation of written science.

While negative modelling results are under-reported, positive modelling results should not be over-interpreted. The fact that a model reproduces a collective pattern does not imply it is necessarily the best or even a good model of the data. Most scientists are aware of this distinction between sufficiency and necessity, but for collective animal behaviour, this point is particularly important. The importance arises as a direct consequence of the capability of theory-driven models to produce global patterns independent of model assumptions. In collective behaviour, most theory-driven models are ‘sufficient’ to produce realistic patterns of group-level behaviour of real animals, but none of the models is ‘necessary’: alternative models based on different assumptions often perform equally well and match the data to a similar precision. Thus, the fact that one particular data-driven model also matches the data does not in itself constitute additional support for the model, over and above that already established by comparison with the data at an individual level. Only if the model is falsified by simulation (if the simulation does not reproduce the global pattern observed in the data) do we really gain additional understanding of the system as a whole.

It is here that model selection approaches can be useful. Once we have a set of potential models, we can use the Bayes factor to determine which gives the best explanation of the data, using the minimum number of necessary parameters. We applied such model selection to prawn collective motion and were able to establish the importance of non-local or non-Markovian interactions. A similar approach has been proposed by Weitz and co-workers, who suggest that models should be ‘enunciated’ and translated into quantitative terms in advance of comparison to individual and group level experiments [68]. In practice, model selection requires that we have already come quite far in understanding what is going on in a system. Computational limitations mean that we should have a limited number of models to test between. This necessarily implies that we must rely on a combination of data-mining—by which we mean plotting behavioural response data in various ways to identify patterns—and ‘biological intuition’ in order to produce a set of models to select between.

As well as requiring a set of models to select between, we need a criterion on which to make our selection. This may actually be easier to define at the local than at the global level. For example, we can measure the probability of turning left or right as a function of the animals' local environment and compare this with several models. The behaviour of individual animals can be further quantified in terms of their speed, their direction of movement, their probability to perform a specific action, etc. At the global level, the challenge is to quantify the form of a fish school or a flock of birds or a termite nest. Many properties can be measured, but it is difficult to discern between those that are actually relevant for understanding the group behaviour. As we collect more group level data, we envisage an increasing understanding of the relevant metrics in different cases.

Collective animal behaviour research is entering an exciting stage where elegant models are being confronted with detailed data of how animals move and interact. As a result, we have seen ‘messier’ models at an individual level, and new challenges as to how we explain the patterns these produce at the global level. Our hope is that through repeated revolutions of the modelling cycle we describe here, applied to a wide range of systems, we will provide a better understanding of how individual interactions produce collective dynamics.
